# Prevalence and Determinants of Metabolic Syndrome among Adults in a Rural Area of Northwest China

**DOI:** 10.1371/journal.pone.0091578

**Published:** 2014-03-10

**Authors:** Yaling Zhao, Hong Yan, Ruihai Yang, Qiang Li, Shaonong Dang, Yuying Wang

**Affiliations:** 1 Department of Epidemiology and Biostatistics, School of Public Health, Xi'an Jiaotong University Health Science Center, Xi’an, Shaanxi, People’s Republic of China; 2 Department of Cardiovascular Diseases, Hanzhong People’s Hospital, Hanzhong, Shaanxi, People’s Republic of China; 3 Department of Physiology and Pathophysiology, Xi'an Jiaotong University Health Science Center, Xi’an, Shaanxi, People’s Republic of China; Tulane School of Public Health and Tropical Medicine, United States of America

## Abstract

**Objectives:**

To evaluate the prevalence and determinants of metabolic syndrome (MetS) among adults in a rural area of Northwest China.

**Methods:**

A population-based cross-sectional study was conducted in 2010 among adults aged 18 to 80 years in rural areas of Hanzhong, in Northwest China. Interview, physical and clinical examinations, and fasting blood glucose and lipid measurements were completed for 2990 adults. The definitions of MetS proposed by the Third Report of the National Cholesterol Education Program Expert Panel (Adults Treatment Panel III, ATP III) and the International Diabetes Federation (IDF), and the modified ATP III definition for Asian population were used and compared. Proportions were adjusted for age and sex.

**Results:**

The prevalence of MetS was 7.9%, 10.8% and 15.1% according to ATP III, IDF and modified ATP III criteria, respectively. Agreement between ATP III and IDF criteria and that between ATP III and modified ATP III criteria were moderate (Kappa* = *0.52 and 0.64, respectively), whereas agreement between IDF and modified ATP III criteria was good (Kappa* = *0.83). The prevalence of MetS increased with age, and was higher in women than in men (10.4% versus 5.4%, 13.6% versus 8.1% and 17.4% versus 12.8%, according to ATP III, IDF and modified ATP III criteria, respectively). The most common MetS component was high blood pressure. Having family history of hypertension, lack of physical activity, high economical level, overweight and obesity were positively associated with MetS.

**Conclusions:**

MetS is prevalent among rural adults in Northwest China and high blood pressure is the most common MetS component. Prevention and treatment of hypertension and MetS should be a public health priority to reduce cardiovascular diseases in rural areas of Northwest China. More attention should be given to the elderly, women, people with family history of hypertension and obese people who are at high risk of MetS.

## Introduction

Metabolic syndrome (MetS) is a complex of interrelated risk factors for cardiovascular disease and diabetes, including elevated fasting glucose, elevated blood pressure, elevated triglycerides (TG) levels, reduced high-density lipoprotein cholesterol (HDL-C) levels, and central obesity [1]. People with MetS are at twice the risk for cardiovascular disease compared with those without the syndrome, and MetS also raises the risk for type 2 diabetes about 5-fold [2], [3]. In addition, persons with MetS are at increased risk of all-cause and cardiovascular disease mortality [4]. Studies show that MetS has a rising prevalence worldwide and it is now both a public health and a clinical problem [1], [2].

With rapid economic progress and associated demographic and epidemiologic transitions in China, cardiovascular diseases have been the leading cause of both morbidity and mortality, responsible for 41% of all annual deaths, and the increase in cardiovascular disease mortality in rural residents is greater than that in urban citizens [5]. Understanding the prevalence and determinants of MetS is critical for allocating health care resources and reducing the increased burden of cardiovascular diseases in China. Some studies have described the prevalence of MetS in the Chinese population [6]–[17]. However, most of them focused on the population in urban [7]–[10], [17], or in the rural areas of East China [11], [12], South China [13], [14] or Northeast China [16]. Little information is available about MetS in the rural areas of Northwest China, which is relatively poor and less developing compared with coastal, eastern and southern regions of China. Only one study by Yi et al. [15] has been reported among rural adults in Ningxia, Northwest China. Ningxia is a Hui ethnicity autonomous region with Hui lifestyle and cultural traditions.

To better understand the epidemiology of MetS in the rural population in Northwest China, in this study, we analyzed the prevalence and determinants of MetS among adults in rural areas of Hanzhong, Shaanxi Province, Northwest China, which is mainly ethnic Han Chinese. Since the prevalence estimates of MetS may differ depending on the definition used [18], we reported and compared the prevalence of MetS using three definitions: one was proposed by the Third Report of the National Cholesterol Education Program Expert Panel on Detection, Evaluation, and Treatment of High Blood Cholesterol in Adults (Adult Treatment Panel III, ATP III, 2001) [19], [20], another was proposed by the International Diabetes Federation (IDF, 2005) [21], [22], the third was the modified ATP III (2005) definition for Asian population proposed by the American Heart Association and the National Heart, Lung, and Blood Institute [23] (Table 1). For the Chinese population, the modified ATP III criteria is the same as the common definition proposed by the International Diabetes Federation Task Force on Epidemiology and Prevention, the National Heart, Lung, and Blood Institute, the American Heart Association, the World Heart Federation, the International Atherosclerosis Society, and the International Association for the Study of Obesity in 2009 [1].

**Table 1:** Diagnosis criteria of metabolic syndrome used in the current study.

MetS component	ATP III criteria [19], [20]	IDF criteria [21], [22]	Modified ATP III criteria [23]
To be identified as Mets	Any three or more of the following five components	Central obesity plus any two other factors	Any three or more of the following five components
Waist circumference			
Men	>102 cm	≥90 cm for Chinese men	≥90 cm for Asian men
Women	>88 cm	≥80 cm for Chinese women	≥80 cm for Asian women
TG	≥1.70 mmol/L (150 mg/dL)	≥1.70 mmol/L (150 mg/dL) mg/dL or specific treatment for this lipid abnormality	≥1.70 mmol/L (150 mg/dL) or drug treatment for elevated TG
HDL-C			
Men	<1.03 mmol/L (40 mg/dL)	<1.03 mmol/L (40 mg/dL) in males or specific treatment for this lipid abnormality	<1.03 mmol/L (40 mg/dL) in men or drug treatment for reduced HDL-C
Women	<1.30 mmol/L (50 mg/dL)	<1.30 mmol/L (50 mg/dL) in women, or specific treatment for this lipid abnormality	<1.30 mmol/L (50 mg/dL) in women or drug treatment for reduced HDL-C
Blood pressure	≥130/85 mm Hg	SBP ≥130 or DBP ≥85 mm Hg, or treatment of previously diagnosed hypertension	≥130 mm Hg SBP or ≥85 mm Hg DBP or on antihypertensive drug treatment in a patient with a history of hypertension
Fasting glucose	≥6.1 mmol/L (110 mg/dL)	≥5.6 mmol/L (100 mg/dL), or previously diagnosed type 2 diabetes	≥5.6 mmol/L (100 mg/dL) or drug treatment for elevated glucose

MetS: metabolic syndrome; TG: triglycerides; HDL-C: high-density lipid cholesterol; SBP: systolic blood pressure; DBP: diastolic blood pressure.

## Methods

### Study population and Data Collection

A population-based cross-sectional study was conducted in the rural areas of Hanzhong, Shaanxi Province, in Northwest China in October 2010 to November 2010, to estimate the cardiovascular disease risk factors and epidemic of hypertension and MetS among rural adults. The participants were restricted to people who had been living at the study sites for at least one year prior to the surveys.

Stratified randomized cluster sampling method was used. There are nine townships in the study area, and about 17 (15 to 36) villages in each township region. We stratified nine strata according to the township, that is, each township was a stratum and one village (cluster) was randomly chosen from each township. Using residential registration data, all the available and eligible adults in the chosen villages were informed of and invited to participate in the survey several days before the survey. In each chosen village, about 330 adults who consented and came to the clinic of the village doctor, where the interview and physician examination were conducted, on the survey day were chosen as subjects of the survey. Data was collected by trained doctors and nurses from Hanzhong People’s Hospital, and several graduate students from Xi'an Jiaotong University College of Medicine also participated in data collection.

Information was collected by interview on age, sex, level of education, marital status, life habits such as occupational physical activity, smoking and drinking, history of hypertension, diabetes and dyslipidemia (elevated TG and reduced HDL-C), use of antihypertensive medications, antidiabetic medications, and medications for dyslipidemia, family economic level, and family history of hypertension. Blood pressure, height, weight, and waist circumference were measured during the physician examination. Weight and height were measured with participants standing without shoes or heavy outer garments, from which body mass index (BMI, kg/m^2^) was calculated. Waist circumference was measured in the erect position at the midpoint between the lowest rib and the superior border of the iliac crest. Using the World Health Organization (WHO) criteria, BMI was categorized into four groups as underweight (BMI<18.5), normal weight (18.5≤BMI<25.0), overweight (25.0≤BMI<30.0) and obesity (BMI≥30.0).

Participants who smoked at least 1 cigarette per day for more than 6 months continuously or cumulatively and did not quit smoking were defined as current smokers [24]. Participants who smoked more than 6 months in their lifetime, but were not smoking at the time of interview were defined as former smokers. Participants who had never smoked a cigarette or who smoked fewer than 6 months in their lifetime were defined as never smokers. We also classified participants into five categories according to their current smoking intensity: non-smoking, ≤5 cigarettes per day, 6 to 10 cigarettes per day, 11 to 20 cigarettes per day and more than 20 cigarettes per day. The cumulative cigarette dose, pack-years of smoking was also calculated as: pack-years  =  (pack per day) * (years smoked). And we classified the participants into four smoking groups: never, ≤7.5 pack-years, 7.6 to 20.0 pack-years, and >20.0 pack-years [25]. Participants were asked about alcohol consumption (including grape wine, rice wine, beer and liquor) within the last year, and were classified into three categories: non-drinkers, moderate drinkers and heavy drinkers. Participants who did not consume any type of alcohol or consumed less than 1 drink per month were defined as non-drinkers. Participants who consumed ≥1 drink any type of alcohol per month were defined as drinkers [24], [26]. Drinkers were further classified as moderate and heavy according to the reference in the Dietary Guidelines for Chinese (≤25 g/d alcohol for men and ≤15 g/d alcohol for women) [27]. Men who consumed ≤25 g/d alcohol and women who consumed ≤15 g/d alcohol were classified as moderate drinkers, and men who consumed >25 g/d alcohol and women who consumed >15 g/d alcohol were classified as heavy drinkers.

Age at interview was categorized in five year intervals. Education status was classed into illiterate, elementary, middle school, and high school and above. Occupational physical activity level was classed into low, moderate and high according to time spent on farm labor or other heavy manual labor: participants who hardly ever did farm labor or other heavy manual labor in the past year were categorized as low occupational physical activity level; participants who sometimes did farm labor or other heavy manual labor were categorized as moderate occupational physical activity level; and participants who often did farm labor or other heavy manual labor were categorized as high occupational physical activity level. To identify the family economic status of the participants, a wealth index based on communication tools, transportation tools, sources of water, and monthly incomes and expenses of the whole family was established using principal component analysis. The first principal component was selected as the wealth index. And the participants’ family economic level was categorized into three groups as low, moderate and high, according to the tertiles of the wealth index [28].

Blood pressure was measured by trained doctors from Hanzhong People’s Hospital, after the subject had rested for at least 5 min, using a standard mercury sphygmomanometer with the participant in the sitting position. Blood pressure was recorded to the nearest 2 mm Hg. Two blood pressure measurements with a two-min interval were obtained, and the mean of two readings was used as blood pressure value. At the time of interview and physician examination, a total of 7 ml overnight fasting blood was collected from each subject using venipuncture by a qualified nurse. Blood sample was drawn into an EDTA containing vacutainer tube and centrifuged within 1 h of collection to measure serum lipids and glucose. Plasma and red blood cells were separated and frozen at –20°C. All specimens were transported on dry ice to the laboratory and stored at –70°C before analyses. The fasting time was verified before blood sample collection and participants who had not fasted for at least 8 hours did not have their blood drawn. Serum glucose, HDL-C and TG levels were analyzed enzymatically using reagents from the Sekisui Chemical Co., Ltd, Japan, using an automatic chemistry analyzer (Hitachi 7080, Tokyo, Japan). Both inter- and intra- assay of variation (coefficient of variation, CV) were less than 3.5% for glucose, TG and HDL-C.

### Statistical Analyses

The Complex Samples Procedure of SPSS 13.0 for Windows (SPSS Inc., Chicago, Illinois, USA) was used for statistical analyses, accounting for township strata and village clusters. All statistical tests were two-tailed, and statistical significance was set at P<0.05. Continuous variables were presented as mean values. Categorical variables were presented as frequencies. We calculated the prevalence of MetS according to the APT III, IDF and modified ATP III criteria. The Kappa statistic was used to determine the agreement between the three criteria. The prevalence of individual component of MetS was calculated based on the modified ATP III criteria. And the prevalence of MetS and individual components of MetS were adjusted for age and/or sex, according to the 2010 Chinese National Census population distribution except for age-specific and/or sex-specific percentages. Differences between means were compared using General Linear Models. Chi-square tests were used to compare frequencies. Logistic Regression Model was used to evaluate the association between MetS (dependent variable) and associated risk factors.

### Ethics Statement

The study complied with the Declaration of Helsinki and was reviewed and approved by the Ethics Committee of Xi’an Jiaotong University College of Medicine and written informed consent had been obtained from the study participants.

## Results

### General characteristics of the study population

A total of 3031 residents aged 18 to 80 years were investigated. 2990 participants who had complete interview and blood sample data were included in the analyses of MetS. Forty-one participants who had no blood sample data, including 20 ones refused to draw blood sample and 21 ones did not fast overnight, were excluded. The socio-demographic characteristics, lifestyle factors, family history of hypertension, average BMI, waist circumference, blood pressure, TG, HDL-C and fasting plasma glucose of these participants are shown in Table 2. 1035 (34.6%) participants were men and 1955 (65.4%) ones were women. The mean age of the participants was 50.6 (SE 1.0) years. Most (99.8%) participants were ethnic Han Chinese and 54.9% of them finished middle school education or above. 91.4% participants were married, 1.5% were unmarried and 7.1% were divorced, separate or widowed. 21.9% participants were current smokers and 32.8% were current drinkers. 35.0% participants had family history of hypertension. 51.2% women were postmenopausal. The mean BMI was 22.9 (SE 0.1). Rates of underweight, normal weight, overweight and obesity were 6.4%, 70.8%, 21.2% and 1.6%, respectively. The mean waist circumference was 81.3 (SE 0.5) cm for men and 77.9 (SE 0.6) cm for women. The mean systolic blood pressure and diastolic blood pressure was 132.1 (SE 1.5) mm Hg and 79.1 (SE 0.9) mm Hg, respectively. The mean TG, HDL-C and fasting plasma glucose was 1.59 (SE 0.04) mmol/L, 1.51 (SE 0.03) mmol/L and 5.15 (SE 0.08) mmol/L, respectively. No statistically significant differences were detected between the 2990 subjects with complete data and those 41 ones without blood sample data.

**Table 2:** Characteristics of the population in the study.

Characteristics	Men (n = 1035)	Women (n = 1955)	Total (n = 2990)	P value^*^
Age, years, mean (SE)	51.6 (1.1)	50.1 (0.9)	50.6 (1.0)	<0.001
Han ethnic group (%)	99.8	99.8	99.8	0.846
Level of education (%)				0.005
Illiterate	5.8	17.8	13.6	
Elementary	30.6	32.0	31.5	
Middle School	47.8	41.0	43.4	
High School and above	15.8	9.2	11.5	
Marital status (%)				<0.001
Married	90.6	91.8	91.4	
Unmarried	3.1	0.7	1.5	
Divorced, separate, widowed	6.3	7.5	7.1	
Smoking status (%)				<0.001
Never	26.0	98.5	73.3	
Former	12.5	0.6	4.8	
Current	61.5	0.9	21.9	
Alcohol drinking (%)				<0.001
Non-drinkers	40.1	81.3	67.2	
Moderate drinkers	43.2	16.7	25.8	
Heavy drinkers	16.7	2.0	7.0	
Family history of hypertension (%)	33.2	36.0	35.0	0.349
Postmenopausal (%)	-	51.2	-	
BMI, kg/m^2^, mean (SE)	22.9 (0.2)	22.9 (0.1)	22.9 (0.1)	0.972
Underweight (BMI<18.5, %)	6.5	6.3	6.4	0.083
Normal (18.5≤BMI<25.0, %)	67.8	72.4	70.8	0.084
Overweight (25.0≤BMI<30.0, %)	23.9	19.8	21.2	0.081
Obesity (BMI≥30.0, %)	1.8	1.5	1.6	0.276
Waist circumference (cm)	81.3 (0.5)	77.9 (0.6)	79.1 (0.6)	<0.001
SBP (mm Hg)	132.5 (1.8)	131.89 (1.4)	132.1 (1.5)	0.542
DBP (mm Hg)	80.2 (0.9)	78.5 (0.9)	79.1 (0.9)	0.007
TG (mmol/L)	1.65 (0.08)	1.55 (0.04)	1.59 (0.04)	0.334
HDL-C (mmol/L)	1.43 (0.03)	1.54 (0.03)	1.51 (0.03)	<0.001
Fasting glucose (mmol/L)	5.17 (0.11)	5.14 (0.06)	5.15 (0.08)	0.668

BMI: body mass index; SBP: systolic blood pressure; DBP: diastolic blood pressure; TG: triglycerides; HDL-C: high-density lipid cholesterol.

P value for the comparison between men and women.

### Prevalence of MetS

The prevalence of MetS defined by the ATP III, IDF and modified ATP III criteria is presented in Table 3. The age- and sex- adjusted prevalence and the 95% confidence interval (95% CI) of prevalence of MetS, according to the ATP III, IDF and modified ATP III criteria, among participants aged 18 to 80 years were 7.9% (4.0%–17.8%), 10.8% (6.5%–20.5%), and 15.1% (9.8%–24.3%), respectively. And the age- and sex- adjusted prevalence and 95% CI among participants aged 35 to 80 years were 10.4% (5.8%–19.5%), 14.9% (9.7%–23.3%), and 20.2% (14.0%–28.4%), respectively.

**Table 3:** Prevalence of metabolic syndrome among the study population.

Characteristics	ATP III criteria			IDF criteria			Modified ATP III criteria		
	Men (n = 1035)	Women (n = 1955)	Total (n = 2990)	Men (n = 1035)	Women (n = 1955)	Total (n = 2990)	Men (n = 1035)	Women (n = 1955)	Total (n = 2990)
	% (95% CI)	% (95% CI)	% (95% CI)	% (95% CI)	% (95% CI)	% (95% CI)	% (95% CI)	% (95% CI)	% (95% CI)
Overall (18–80 years)	5.4 (2.2–14.6)*	10.4 (5.9–21.1)*	7.9 (4.0–17.8)^ †^	8.1 (3.9–18.4)*	13.6 (9.2–22.8)*	10.8 (6.5–20.5)^ †^	12.8 (7.5–21.5)*	17.4 (12.2–27.2)*	15.1 (9.8–24.3)^ †^
Overall (35–80 years)	7.2 (3.3–15.9)*	13.8 (8.5–23.2)*	10.4 (5.8–19.5)^ †^	11.0 (6.0–20.7)*	19.0 (13.5–26.1)*	14.9 (9.7–23.3)^ †^	16.3 (10.3–25.0)*	24.1 (17.9–31.9)*	20.2 (14.0–28.4)^ †^
Age group (years)									
18.0–24.9	0.0 (0.0–0.0)	2.4 (0.3–16.8)	1.2 (0.1–8.3)^‡^	0.0 (0.0–0.0)	2.4 (0.3–16.8)	1.2 (0.1–8.3)^‡^	0.0 (0.0–0.0)	2.4 (0.3–16.8)	1.2 (0.1–8.3)^‡^
25.0–29.9	3.2 (0.3–29.4)	3.7 (0.8–15.2)	3.5 (0.5–22.3)^‡^	4.8 (0.7–26.5)	3.7 (0.8–15.2)	4.2 (0.7–20.9)^‡^	12.5 (4.7–29.2)	3.7 (0.8–15.2)	8.1 (2.8–22.2)^‡^
30.0–34.9	3.8 (0.9–14.3)	4.2 (1.2–13.7)	4.0 (1.1–14)^‡^	6.5 (1.6–22.5)	3.0 (0.6–13.6)	4.7 (1.1–18.1)^‡^	11.1 (3.9–27.8)	5.5 (1.5–18.1)	8.4 (2.7–23.0)^‡^
35.0–39.9	4.0 (1.6–9.8)	5.3 (1.2–21.1)	4.6 (1.4–15.3)^‡^	9.4 (5.1–16.6)	9.8 (5.8–16.0)	9.6 (5.4–16.3)^‡^	13.4 (9.2–19.1)	12.5 (7.3–20.8)	13.0 (8.3–19.9)^‡^
40.0–44.9	8.3 (3.5–18.2)	5.4 (2.8–10.2)	6.9 (3.2–14.2)^‡^	9.9 (5.7–16.6)	9.3 (5.8–14.6)	9.6 (5.8–15.6)^‡^	13.7 (7.5–23.6)	12.6 (7.7–19.9)	13.2 (7.6–21.8)^‡^
45.0–49.9	8.7 (4.6–15.9)	13.9 (8.8–21.3)	11.3 (6.7–18.6)^‡^	11.4 (6.4–19.3)	17.8 (13.7–22.9)	14.6 (10.0–21.1)^‡^	16.0 (10.7–23.2)	22.8 (18.1–28.2)	19.3 (14.3–25.6)^‡^
50.0–54.9	8.1 (5.3–12.1)	16.0 (11.0–22.7)	11.9 (8.1–17.2)^‡^	13.6 (9.4–19.1)	20.5 (13.7–29.5)	16.9 (11.5–24.2)^‡^	19.6 (12.5–29.4)	26.9 (19.9–35.2)	23.2 (16.1–32.2)^‡^
55.0–59.9	6.5 (2.0–18.9)	20.8 (14.7–28.6)	13.6 (8.3–23.7)^‡^	14.2 (8.3–23.1)	26.9 (20.7–34.2)	20.5 (14.4–28.6)^‡^	19.6 (10.4–33.9)	32.1 (27–37.6)	25.8 (18.6–35.8)^‡^
60.0–64.9	7.3 (2.3–20.4)	23.9 (17.8–31.3)	15.4 (9.9–25.8)^‡^	12.0 (4.7–27.4)	37.0 (28.4–46.5)	24.3 (16.4–36.8)^‡^	19.7 (12.3–30.1)	44.2 (36–52.7)	31.7 (23.9–41.2)^‡^
65.0–80.0	8.4 (3.4–19.1)	22.8 (12.1–38.7)	15.7 (7.9–29.1)^‡^	7.8 (1.6–29.8)	26.8 (18.7–36.8)	17.5 (10.4–33.4)^‡^	16.0 (11.8–21.3)	36.1 (25–48.9)	26.3 (18.5–35.4)^‡^

95% CI: 95% confidence interval.

^*^Age- adjusted percentages for men or women. ^†^Age- and sex- adjusted percentages. ^‡^ Sex-adjusted percentages for each age group. Adjustment was conducted with the 2010 Chinese National Census Population by the direct method.

**Figure 1:**
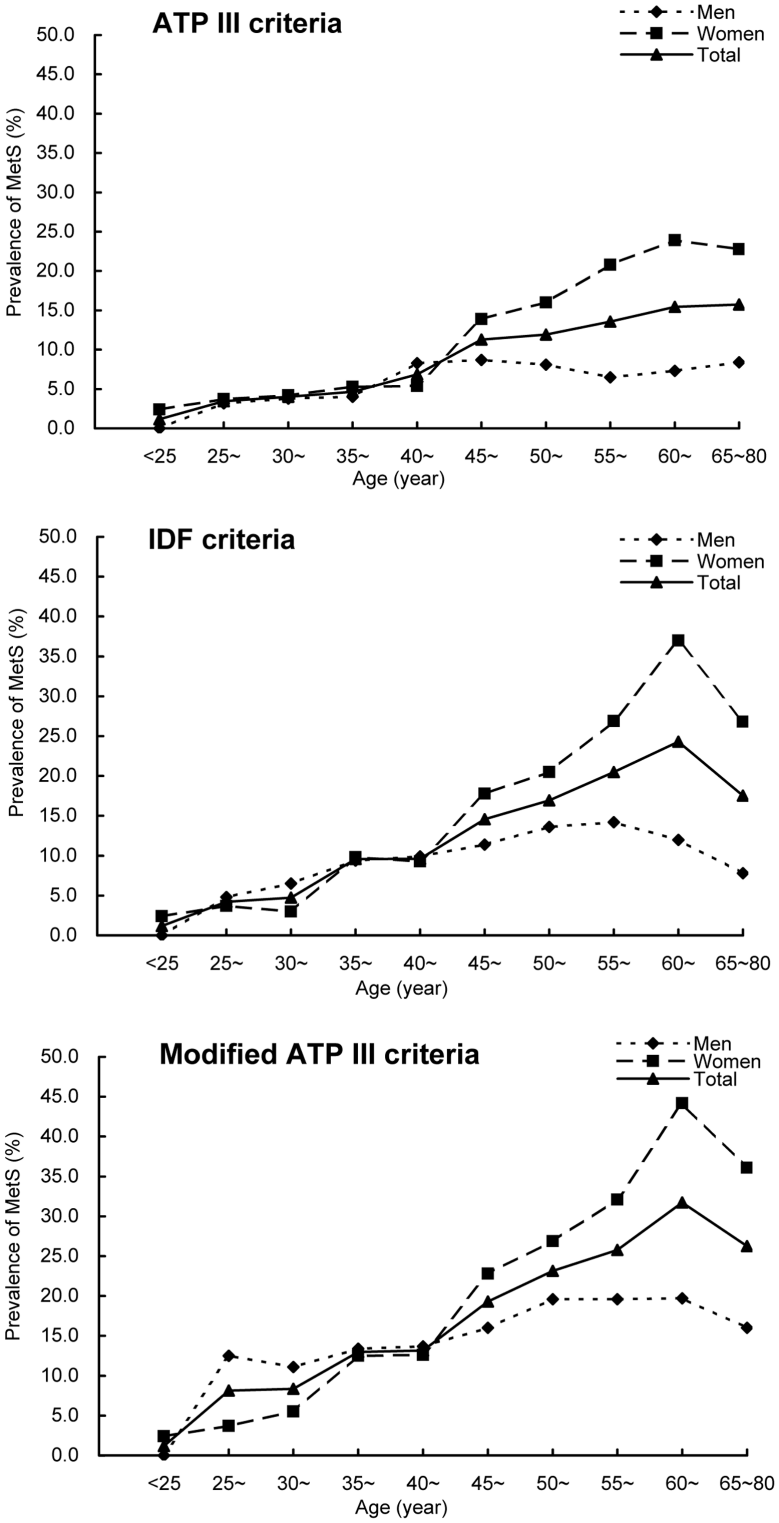
Prevalence of MetS among adults aged 18 to 80 years in the study area.

The age-standardized prevalence of Mets according to the ATP III, IDF and modified ATP III criteria for men aged 18 to 80 years were 5.4% (95% CI: 2.2%–14.6%), 8.1% (95% CI: 3.9%–18.4%) and 12.8 (95% CI: 7.5%–21.5%), respectively, and the prevalence for women aged 18 to 80 years were 10.4% (95% CI: 5.9%–21.1%), 13.6% (95% CI: 9.2%–22.8%), and 17.4% (95% CI: 12.2%–27.2%), respectively. The age-specific prevalence for women according to all those three criteria were higher than those for men among adults aged 45.0 years or older (P<0.01), however, among adults younger than 45.0 years old, the prevalence for women were higher or lower than, or had no statistical difference with the prevalence for men, depending on the MetS criteria and age groups (Table 3, Figure 1). And the sex-standardized prevalence of MetS according to those three criteria all increased with increasing age until the age of 64.9 and decreased thereafter (P<0.001, Table 3, Figure 1). In women, the 45.0–64.9 years age groups showed a marked increase in the prevalence of MetS, and up to the highest in 60.0–64.9 years age group (Table 3, Figure 1). More than 20% women aged 55.0 years or older were identified as having MetS by either diagnosis criteria. In men, the prevalence of MetS also increased with age increasing, however, the increase in prevalence between age groups was not statistically significant (Table 3, Figure 1).

### Agreement between the three MetS definitions

**Figure 2:**
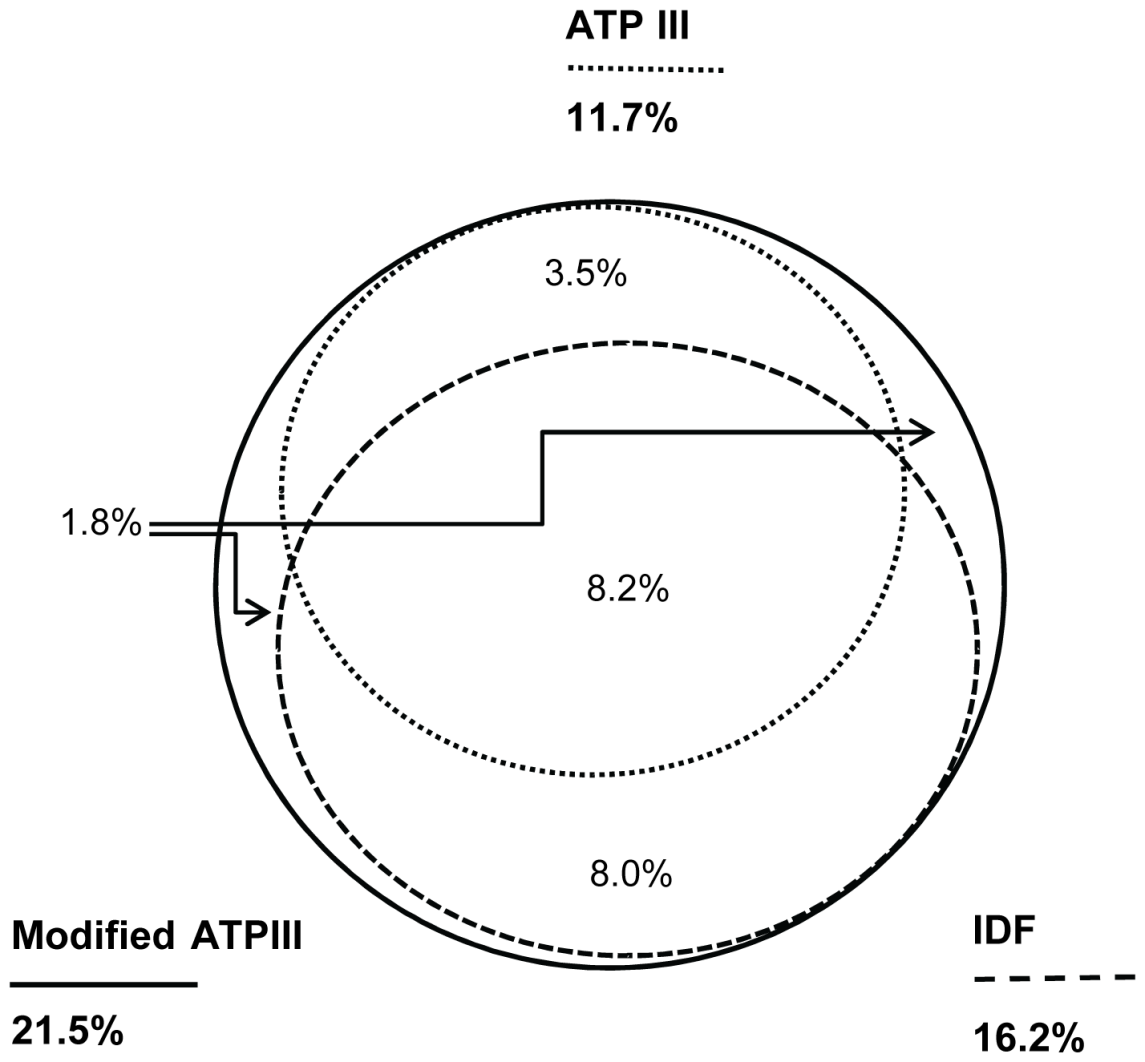
Overall crude prevalence of MetS according to ATP III, IDF and modified ATP III criteria.

Figure 2 shows the unadjusted overall prevalence of MetS according to the ATP III, IDF and modified ATP III criteria. Only 8.2% (95% CI: 6.3%–10.6%) participants were identified as MetS by all three criteria. The levels of agreement of the ATP III criteria with the IDF and modified ATP III criteria were moderate, with Kappa statistics of 0.52 and 0.64, respectively (both P<0.001). The agreement between the IDF and modified ATP III criteria was good, with Kappa statistics of 0.83 (P<0.001). And from the Figure 1 we found that the sex-adjusted age-specific prevalence of MetS according to the ATP III criteria was lower than the prevalence according to the IDF and modified ATP III criteria, and the prevalence according to the modified ATP III was the highest (all P<0.05 for the comparisons between the age-specific prevalence according to three criteria among 35.0 to 80.0 years age groups).

### Prevalence of individual components of MetS

The prevalence of individual components of MetS, by the modified ATP III criteria, among the study population is presented in Table 4. High blood pressure (≥130/85 mm Hg) was the most common MetS component in both men and women. The age- and sex- adjusted prevalence of high blood pressure was 39.0%. And the age- adjusted prevalence was 42.7% for men and 35.3% for women. Elevated TG was the second and third highest MetS component in men and women (32.3% versus 24.8%), respectively. Central obesity was the second common MetS component (30.2%) in women. The triad of high blood pressure, elevated TG and central obesity accounted for about half of the MetS in this population (48.0% in men, 56.7% in women, and 52.3% in total). The prevalence of elevated TG, high blood pressure and elevated fasting plasma glucose among men were higher than those among women (all p<0.001), and the prevalence of the other two components of MetS, central obesity and reduced HDL-C among men were lower than those among women (both P<0.001). 21.9% participants had central obesity according to the modified ATP III criteria’s ethnic specific cut point (90 cm for Chinese men and 80 cm for Chinese women), however, only 5.5% participants met the criteria for central obesity according to the ATP III criteria’s cut point (102 cm for men and 88 cm for women). Of the modified ATP III criteria 34.4% participants had none of its components. 65.6% had at least one component, and 1.0% had all five components (Table 5).

**Table 4:** Prevalence of individual components of metabolic syndrome.

Component of metabolic syndrome	Men (n = 1035)‡	Women (n = 1955)‡	Total (n = 2990)^§^	P value¶
	% (95% CI)	% (95% CI)	% (95% CI)	
Central obesity				
>102 cm for men, >88 cm for women*	1.5 (0.3–6.2)	9.5 (4.9–18.4)	5.5 (2.6–12.2)	<0.001
≥90 cm for men, ≥80 cm for women^†^	13.7 (7.6–24.4)	30.2 (21.1–41.8)	21.9 (14.3–33.0)	<0.001
Elevated TG^†^	32.3 (21.9–49.0)	24.8 (17.7–36.0)	28.6 (19.8–42.6)	<0.001
Reduced HDL-C^†^	8.8 (3.9–23.0)	22.3 (13.6–35.0)	15.5 (8.7–28.9)	<0.001
High blood pressure (≥130/85mm Hg)^†^	42.7 (30.5–58.1)	35.3 (29.8–42.7)	39.0 (30.1–50.5)	<0.001
Elevated fasting plasma glucose^†^	16.2 (8.3–34.4)	12.4 (8.1–21.5)	14.3 (8.2–28.0)	<0.001

TG: triglycerides; HDL-C: high-density lipid cholesterol; 95% CI: 95% confidence interval.

^*^ATP III criteria. ^†^modified ATP III criteria.

‡ Age- adjusted percentages for men or women. ^§^Age- and sex- adjusted percentages. Adjustment was conducted with the 2010 Chinese National Census Population by the direct method.

¶ P value for the comparison between men and women.

**Table 5:** Percentage of participants with individual components of metabolic syndrome by the ATP III modified definition.

Number of individual component	Men (n = 1035)*	Women (n = 1955)*	Total (n = 2990)^†^	‡ P value
	% (95% CI)	% (95% CI)	% (95% CI)	
0	34.2 (19.6–50.7)	34.7 (25.2–44.9)	34.4 (22.3–47.8)	0.883
1	34.3 (22.4–49.6)	30.9 (23.6–39.5)	32.6 (23.0–44.6)	0.287
2	18.7 (11.3–31.6)	17.1 (11.6–27.5)	17.9 (11.5–29.6)	0.568
3	9.8 (5.1–19.1)	11.3 (7.4–19.2)	10.6 (6.3–19.1)	0.633
4	2.5 (0.7–10.7)	4.5 (2.6–8.8)	3.5 (1.6–9.8)	0.599
5	0.5 (0.1–3.5)	1.6 (0.7–3.9)	1.0 (0.4–3.7)	0.668

95% CI: 95% confidence interval.

^*^Age- adjusted percentages for men or women. ^†^Age- and sex- adjusted percentages. Adjustment was conducted with the 2010 Chinese National Census Population by the direct method.

‡ P value for the comparison between men and women.

### Risk Factors for MetS

Results of Complex Samples Logistic Regression Model analysis on the risk factors for MetS according to the Modified ATP III criteria are presented in Table 6. Women, older, divorced, separate, widowed, having family history of hypertension, overweight (25.0≤BMI<30.0), obesity (BMI ≥30.0), low occupational physical activity level, and higher family economical level were risk factors for MetS. Compared with men, the odds ratio (OR) of women was 2.09 (95% CI: 1.45–3.03). And compared with participants younger than 25.0 years olds, the ORs increased with age increasing, from 3.73 (95% CI: 0.08–167.23) of 25.0–29.9 years olds to 35.26 (95% CI: 1.19–45.53) of 60.0–64.9 years olds. Although the OR of the participants aged 65.0 years and older had a decrease, it still was 20.95 (95% CI: 1.81–44.89). The OR of divorced, separate or widowed was 1.39 (95% CI: 1.09–1.97) and OR of having family history of hypertension was 1.69 (95% CI: 1.46–1.95). Overweight (OR: 5.71; 95% CI: 4.15–7.85) and obesity (OR: 26.86; 95% CI: 13.32–54.14) were risk factors for MetS. Underweight was a protective factor for MetS (OR: 0.13; 95% CI: 0.04–0.42). Compared with participants who had high occupational physical activity level, those who had low occupational physical activity level were at high risk of Mets, with an OR of 1.71 (95% CI: 1.24–2.36). Compared with participants at moderate family economical level, those at high family economical level were at high risk of MetS (OR: 1.23; 95% CI: 1.01–1.50). High educational level was a protective factor for MetS. Compared with illiterate, the ORs were 0.73 (95% CI: 0.51–0.98) of elementary, 0.71 (95% CI: 0.52–0.97) of middle school and 0.57 (95% CI: 0.60–0.87) of high school and above. No association was found between smoking status (never, former, current smoking) and alcohol drinking (none, moderate, heavy drinkers) and Mets (both P>0.05) (Table 6). We also analyzed the association between smoking and MetS using smoking intensity and cumulative cigarette dose, respectively, as index of smoking instead of smoking status (never, former, current smoking), and analyzed the association between different type of drinking (grape wine, rice wine, beer and liquor) and MetS. Logistic regression analyses showed there was no statistical association between smoking intensity or cumulative cigarette dose and MetS, and no statistical association between type of drinking and MetS (all P>0.05, data not shown).

**Table 6:** Results of logistic regression analysis of risk factors for metabolic syndrome.

Variables	OR (95% CI)	P value
Gender		
Men	1.00 (reference)	
Women	2.09 (1.45–3.03)	<0.001
Age group (years)		
18.0–24.9	1.00 (reference)	
25.0–29.9	3.73 (0.08–167.23)	0.424
30.0–34.9	4.26 (0.30–60.59)	0.208
35.0–39.9	7.73 (0.39–53.12)	0.114
40.0–44.9	7.51 (0.34–63.98)	0.131
45.0–49.9	13.34 (0.47–38.08)	0.075
50.0–54.9	18.81 (1.04–33.77)	0.021
55.0–59.9	27.43 (1.09–61.26)	0.018
60.0–64.9	35.26 (1.19–45.53)	0.015
65.0–80.0	20.95 (1.81–44.89)	0.031
Level of education		
Illiterate	1.00 (reference)	
Elementary	0.73 (0.51–0.98)	0.049
Middle School	0.71 (0.52–0.97)	0.023
High school and above	0.57 (0.60–0.87)	0.014
Marital status		
Married	1.00 (reference)	
Unmarried	0.36 (0.07–1.92)	0.158
Divorced, separate, widowed	1.39 (1.09–1.97)	0.030
Smoking status		
Never	1.00 (reference)	
Former	1.51 (0.98–2.34)	0.128
Current	1.24 (0.63–2.44)	0.461
Alcohol drinking		
Non-drinkers	1.00 (reference)	
Moderate drinkers	0.76 (0.51–1.13)	0.113
Heavy drinkers	0.89 (0.51–1.53)	0.613
Family history of hypertension		
No	1.00 (reference)	
Yes	1.69 (1.46–1.95)	<0.001
BMI category		
Normal weight (18.5≤BMI<25.0)	1.00 (reference)	
Underweight (BMI<18.5)	0.13 (0.04–0.42)	<0.001
Overweight (25.0≤BMI<30.0)	5.71 (4.15–7.85)	<0.001
Obesity (BMI≥30.0)	26.86 (13.32–54.14)	<0.001
Occupational physical activity level		
High	1.00 (reference)	
Moderate	1.05 (0.73–1.52)	0.760
Low	1.71 (1.24–2.36)	<0.001
Family economic level		
Moderate	1.00 (reference)	
Low	1.15 (0.90–1.46)	0.191
High	1.23 (1.01–1.50)	0.016

BMI: body mass index; 95% CI: 95% confidence interval.

We also analyzed the risk factors for MetS, according to the modified ATP III criteria, among men and women separately. Results of separate analyses were similar to results of the overall analysis among 2990 participants: the value of ORs had some differences; however, the direction of ORs was the same. We explored the association between menopause and MetS among women. The result showed there was no statistical association between menopause and MetS when adjusted for age, educational level, marital status, having family history of hypertension, BMI, smoking, drinking, occupational physical activity level and family economical level. We also analyzed the risk factors for Mets according to the ATP III criteria and IDF criteria. The results were similar to the analysis according to the modified ATP III criteria. So, we only present results of the overall analysis according to the modified ATP III criteria.

## Discussion

In this study we have described the prevalence of MetS according to three different criteria among a mainly ethnic Han Chinese rural population in Northwestern China. Adjusted for age and sex according to the 2010 Chinese National Census population distribution, 7.9%, 10.8% and 15.1% rural adults aged 18 to 80 years in Northwest China met the ATP III, IDF and modified ATP III criteria for MetS, respectively.

The prevalence of MetS in this population was higher than the prevalence among rural adults (4.9%, 7.2%, and 7.9%, according to the ATP III, IDF, and modified ATP III, respectively) in East China, during 2004 to 2005 [11], and higher than that among rural adults (4.3% according to the IDF criteria) in South China, in 2002 [14]. And the prevalence of MetS in this population was lower than the prevalence among urban adults (15.9% and 28.4% for men, 26.7% and 35.1% for women, according to the IDF and the modified ATP III criteria, respectively) from a study in Shanghai, East China, in 2008 [10], and lower than that among adults (22.4% according to the IDF criteria) in Northeast China [16]. The prevalence in this population was similar to that of the Han ethnic group (10.3% according to the IDF criteria) and lower than that of the Hui ethnic group (13.7% according to the IDF criteria) aged 25 years or older in the rural area in Ningxia, Northwest China, in 2008 [15]. It was also lower than those of some other countries or areas. Data from a nationally representative sample of the Korean adults showed the prevalence of MetS (ATP III criteria) was 14.4% in 2001 [29]. A study in the USA adults from the 1999 to 2006 National Health and Nutrition Examination Survey reported the prevalence of MetS (ATP III criteria) was 39.9% in rural and 32.8% in urban [30]. Among adults in southeast Iran the prevalence of MetS was 21.0% and 24.8% according to the ATP III and IDF criteria, respectively [31]. Our findings confirmed the trends described by Gu et al., that is the prevalence of MetS varied between different regions in China, in urban was higher than that in rural and in the North was higher than that in the South [6], and that the Chinese population had low prevalence of MetS compared with other ethnic populations.

The agreement between the ATP III and the IDF criteria and the ATP III and the modified ATP III criteria were moderate, and the agreement between the IDF criteria and the modified ATP III criteria was good. Our findings are consistent with previous studies conducted in Chinese adults [8], [10], [25] and other populations, such as Korean [32], Malaysian [33] and Portuguese [34]. Our study showed that the IDF criteria gave higher prevalence estimates than the ATP III criteria did. It is in accordance with the results of other studies in China [7], [8], [11], [25], and studies in the USA [35], Qatar [36], Iran [31], Korea [32] and Portugal [34]. Our study also found that the modified ATP III criteria gave higher prevalence estimates than the estimates according to the ATP III and IDF criteria as other studies in China [8], [10], [11], [25]. These findings are mainly due to the lower blood glucose criterion in the IDF and modified ATP III criteria than that in the ATP III criteria, the lower ethnic-specific waist circumference cut-off points for the Chinese in the IDF and modified ATP III criteria, and the modified ATP III criteria not taking central obesity as a requisite for MetS. So, the modified ATP III criteria may be more suitable for the Chinese population to detect and treatment MetS earlier. However, whether different criteria of MetS differ in their ability to predict cardiovascular events and diabetes is not known. A study in Greece reported that the IDF criteria of MetS appeared to be a better predictor of acute coronary syndrome than the ATP III and modified ATP III criteria [37]. But a study in Sweden showed that the IDF criteria was not superior to the ATP III criteria for prediction of cardiovascular events [38]. To better understand this issue, more studies are needed.

When using the modified ATP III criteria, high blood pressure was the most common MetS component in the study population. It is consistent with the results of other studies in China [6], [12], [17]. However, several studies in the USA and Canada reported that central obesity was the most common component of MetS [39]–[41]. It may be due to that the population blood pressure level and prevalence of hypertension have lowered or remained stable [42]–[44], while the prevalence of obesity has increased and remained at a high level [45]–[47] in the developed countries over the past decades. But blood pressure levels and hypertension in most developing countries are increasing and coming closer to the developed countries [48]. Our previous study showed that the average blood pressure levels and prevalence of hypertension among adults in the rural areas of Hanzhong had increased since 1982, however, awareness, treatment and control rates of hypertension remained low [49]. So, public health programs and practical strategies aimed at prevention and control of hypertension should therefore be prioritized to reduce the occurrence of MetS and cardiovascular diseases in the rural areas of Northwest China.

Our study showed that women were at higher risk of MetS than men. The prevalence for women according to all three criteria were higher than those for men among adults aged 45 years or older. Logistic regression analysis also showed that being women was a risk factor for MetS. Our findings are consistent with results of other studies in China [10], [12], [14], Korea [29], Southeast Iran [31], Qatar [36], Turkey [50], and the Mexican American and African Americans in the USA [51]. It suggests that more attention should be given to women in the prevention and control of MetS and relevant cardiovascular diseases.

Physical inactivity and diet rich in saturated fat and cholesterol may enhance risk for MetS and enhance risk for developing cardiovascular disease in people with MetS [20], [52]–[55]. With the rapid economic progress, China has been experiencing a lifestyle and nutrition transition characterized by an increasing inactivity at work and leisure time and a shift towards high-fat, high-energy-density and low-fiber diet [56]–[59]. And a study in China reported that income was positively associated with less healthy eating behaviors such as consumption of snacks and excessive fried food [60]. Our study also showed that the prevalence of MetS was positively associated with low occupational physical activity level and higher family economical level. So, effective health education programs and practical intervention strategies should be carried out to promote physical activity and to improve the diet behaviors in the Chinese population, especially among the people with higher economic status.

Our study also showed that the prevalence of MetS was positively associated with increasing age, low educational level, divorced, separate, widowed, having family history of hypertension, overweight and obesity. These findings suggest that the elderly, obese people, divorced, separate or widowed people, people with low educational level, and people with family history of hypertension are at high risk of MetS and should be the important target population of prevention and control of MetS.

### Limitations and strengths of the study

Our study has several potential limitations. First, the participants’ family history of MetS, diabetes, stroke, coronary heart disease and other cardiovascular diseases were not collected in the survey. So, we could not analyze the association between these variables and MetS in the study population. However, several studies reported that having family history of diabetes, stroke or coronary heart disease was positively associated with MetS [61]–[64]. Second, in this study, we only collected and analyzed participants’ occupational physical activity data, and did not collect data about their leisure time physical activity. Although a study among women in rural areas of East China reported no association between leisure time physical activity and MetS [25], and leisure time physical activity may not be the main component of physical activity among people who have high levels of occupational physical activity, such as farmers and workers, with the urbanization and the lifestyle change in China, leisure time physical activity may become more and more important for the rural population. So, more studies are needed to collect and analyze detailed data on the quantity and category of occupational and leisure time physical activity among rural population in China. Third, our cross-sectional study design does not allow us to draw any causal inference. Therefore, future studies, especially prospective studies are needed to confirm the association between exposures, including family history of cardiovascular diseases and physical activity at work and leisure time, and MetS among rural population in China. Fourth, the IDF [65] recommended that waist circumference should be measured at the midpoint between the lowest rib and the superior border of the iliac crest, as suggested by the WHO, whereas the ATP III and modified ATP III recommended waist circumference be measured directly above the superior border of the iliac crest as the USA National Institutes of Health (NIH) guidelines specified [23], [66], [67]. In our study, we used the WHO method to measure waist circumference and used the measurements to estimate the prevalence of MetS according to the IDF, ATP III and modified ATP III criteria as other studies did [7], [9], [10], [14], [33]. However, Wang et al. reported that waist circumference measurements based on the NIH method were significantly greater than measurements based on the WHO method among women [67], and Mason et al. reported that measurement site had an influence on the prevalence of abdominal obesity for women (>88 cm, 41.1% based on the WHO method versus 47.0% on the NIH method) [68]. A study in the Canada population showed that adults’ waist circumference measurements based on the NIH method significantly exceeded measurements based the WHO method (0.8 cm for men and 2.2 cm for women), and developed prediction equations to transform waist circumference measures taken at those two different measurement sites [69]. Using their equations to predict the NIH waist circumference measures based on waist circumference measured using the WHO method for adults aged 20 to 79 years [for men, (predicted NIH waist circumference)  =  3.83072 + 0.98613 * (waist circumference measured using WHO method) – 0.03609 * (age); and for women, (predicted NIH waist circumference)  =  3.53771+ 0.98479 * (waist circumference measured using WHO method) + 0.21949 * (x), where x is set to 1 if age is 20 to 39, otherwise x = 0] [69], we predicted the NIH waist circumference and estimated the prevalence of MetS according to the ATP III and modified ATP III criteria in our study population. Results showed both the predicted age- and sex- adjusted prevalence of MetS according to the ATP III and modified ATP III criteria were higher than the prevalence calculated with waist circumference based on the WHO method [8.9% (95% CI: 4.5%–19.3%) versus 7.9% (95% CI: 4.0%–17.8%), according to the ATP III criteria; 16.4% (95% CI: 10.5%–26.5%) versus 15.1% (95% CI: 9.8%–24.3%), according to the modified ATP III criteria). So, using waist circumference measures based on the WHO method may underestimate the prevalence of MetS according to the ATP III and modified ATP III criteria in our study population, although the rationality of using those equations in the Chinese population is not known. Finally, we recognize that our study is mainly confirmatory and selection bias might be present due to over representation of women in our sample, though standardized prevalence might have mitigated such bias.

Despite these limitations the study was done using a relatively large population-based sample of adults from a rural area of Northwest China and should therefore provide important information regarding the burden of MetS in the rural population of similarly aged Northwest Chinese. Moreover, it is well-known that the prevalence estimates of MetS in a population may differ depending on the criteria used. Our study is the first study reporting the prevalence of MetS using the ATP III, IDF and modified ATP III criteria among rural adults in Northwest China. It is convenient for the comparison of the prevalence of the study population with that of other populations using different MetS criteria.

## Conclusions

The MetS is prevalent among rural population, especially among the elderly and women, in Northwest China regardless of the definition of MetS used. High blood pressure is the most common component of MetS in this population. Having family history of hypertension, lack of physical activity, overweight and obesity were risk factors for MetS. Prevention and treatment of hypertension and MetS should be a public health priority to reduce the cardiovascular diseases in rural areas of Northwest China. More attention should be given to the elderly, women, people with family history of hypertension and obese people who are at high risk of MetS.
